# Central focused convolutional neural networks: Developing a data-driven model for lung nodule segmentation

**DOI:** 10.1016/j.media.2017.06.014

**Published:** 2017-06-30

**Authors:** Shuo Wang, Mu Zhou, Zaiyi Liu, Zhenyu Liu, Dongsheng Gu, Yali Zang, Di Dong, Olivier Gevaert, Jie Tian

**Affiliations:** a CAS Key Laboratory of Molecular Imaging, Institute of Automation, Chinese Academy of Sciences, Beijing 100190, China; b Stanford Center for Biomedical Informatics Research (BMIR), Department of Medicine, Stanford University, CA 94305, USA; c University of Chinese Academy of Sciences, Beijing 10 0 049, China; d Guangdong General Hospital, Guangzhou, Guangdong 510080, China; e Beijing Key Laboratory of Molecular Imaging, Beijing 100190, China

**Keywords:** Lung nodule segmentation, Convolutional neural networks, Deep learning, Computer-aided diagnosis

## Abstract

Accurate lung nodule segmentation from computed tomography (CT) images is of great importance for image-driven lung cancer analysis. However, the heterogeneity of lung nodules and the presence of similar visual characteristics between nodules and their surroundings make it difficult for robust nodule segmentation. In this study, we propose a data-driven model, termed the Central Focused Convolutional Neural Networks (CF-CNN), to segment lung nodules from heterogeneous CT images. Our approach combines two key insights: 1) the proposed model captures a diverse set of nodule-sensitive features from both 3-D and 2-D CT images simultaneously; 2) when classifying an image voxel, the effects of its neighbor voxels can vary according to their spatial locations. We describe this phenomenon by proposing a novel central pooling layer retaining much information on voxel patch center, followed by a multi-scale patch learning strategy. Moreover, we design a weighted sampling to facilitate the model training, where training samples are selected according to their degree of segmentation difficulty. The proposed method has been extensively evaluated on the public LIDC dataset including 893 nodules and an independent dataset with 74 nodules from Guangdong General Hospital (GDGH). We showed that CF-CNN achieved superior segmentation performance with average dice scores of 82.15% and 80.02% for the two datasets respectively. Moreover, we compared our results with the inter-radiologists consistency on LIDC dataset, showing a difference in average dice score of only 1.98%.

## 1. Introduction

Lung cancer is the leading cause for cancer related deaths and carrying a dismal prognosis with a 5-year survival rate at only 18% (Siegel et al., 2016). Treatment therapy monitoring and lung nodule analysis (Aerts et al., 2014) using computed tomography (CT) images are important strategies for early lung cancer diagnosis and survival time improvement. In these approaches, accurate lung nodule segmentation is necessary that can directly affect the subsequent analysis results. Specifically, given the fact of growing volumes of clinical imaging data, developing a data-driven segmentation model is of great clinical importance to avoid tedious manual processing and reduce inter-observer variability (Kubota et al., 2011).

**Fig. 1:**
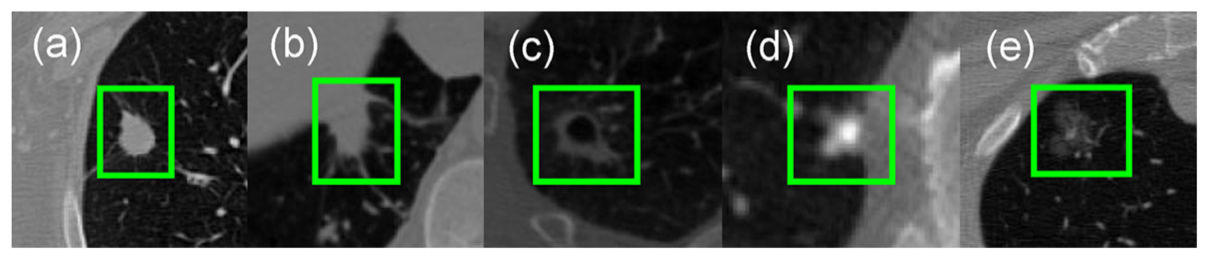
Example images of lung nodules with different locations and shapes in CT: (a) common isolated nodule. (b) juxtapleural nodule. (c) cavitary nodule. (d) calcific nodule. (e) ground-glass opacity (GGO) nodule.

Despite development of approaches for lung nodule segmentation in recent years (Farag et al., 2013; Kubota et al., 2011; Lassen et al., 2015), achieving accurate segmentation performance continues to require attention because of the heterogeneity of lung nodules as shown on CT images (Fig. 1). The presence of similar visual characteristics between nodules and their surroundings poses a technical challenge for developing robust segmentation models. For example, juxtapleural nodules (Fig. 1(b)) have an intensity similar to that of lung wall; thus, they are difficult to distinguish using intensity values only. In addition, cavitary nodules with black hole inside (Fig. 1(c)) and calcific nodules (Fig. 1(d)) are challenging cases because of the intensity dissimilarity within different part of nodules. Similarly, non-solid nodules such as ground-glass opacity (GGO, Fig. 1(e)) are also problematic because a simple morphological operation is not suitable for these cases due to the fact of low intensity contrast in CT data (Dehmeshki et al., 2008).

Intensity-based methods using morphological operation (Diciotti et al., 2011; Messay et al., 2010) and region growing (Dehmeshki et al., 2008; Kubota et al., 2011) have been studied. Energy optimization methods including level set (Farag et al., 2013) and graph cut (Ye et al., 2010) were also researched for lung nodule segmentation. However, the robustness is still problematic especially for segmenting juxtapleural nodules. For example, in morphology-based methods, the morphological template size is difficult to generalize with nodules of various diameters (Kubota et al., 2011). Sophisticated methods can process juxtapleural nodules by applying a shape constraint (Farag et al., 2013; Keshani et al., 2013) or relying on user interactive parameter settings (Messay et al., 2015). However, it may not be applicable for irregular shaped nodules where the shape hypothesis can be violated. In addition, user interactive parameters such as well centralized seed point (Messay et al., 2015) or stroke (Lassen et al., 2015) are difficult to tune for different types of nodules. The limitations of directly applying raw intensity value for segmentation suggest the need of novel solutions for capturing high-level, nodule-sensitive features from CT volumes.

Recently, convolutional neural networks (CNN) have been emerged as powerful tools for learning discriminative feature hierarchies adapted to different vision tasks (Havaei et al., 2016; Shen et al., 2016). Benefiting from the unique feature learning ability from hierarchical network layers, CNN models have shown encouraging results in medical image segmentation tasks (Moeskops et al., 2016; Valverde et al., 2017; Zhang et al., 2015), indicating the usefulness of CNN-based models for medical object segmentation. However, the applicability of developing CNN-based approaches to model heterogeneous lung nodule CT volumes (as seen in Fig. 1) has remained uncertain. In particular, the design of network hierarchy that is capable of capturing both 2-D and 3-D lung nodule features has not been explicitly addressed.

In this study, we investigate the problem of developing a deep hierarchy of convolutional neural networks in the context of lung nodule segmentation. We follow a voxel classification scheme that aims to distinguish nodule voxels from healthy voxels in CT images. In addressing the challenge of analyzing heterogeneous CT data, we propose a central focused convolutional neural networks (CF-CNN) that is adaptive to lung nodule segmentation for various types of nodules. Overall, our technical contributions in this work are four-fold:

1. The proposed CF-CNN model can achieve appealing segmentation performance for a variety of lung nodules especially for juxtapleural nodules without nodule shape hypothesis or user-interactive parameter setting (Fig. 1).
2. We present a two-branch CNN structure to leverage both 3-D features and multi-scale 2-D features. The 3-D-patch branch learns multi-view features from multiple CT slices and the 2-D-patch branch learns multi-scale features through multiple 2-D patches. The multi-scale patch strategy enables the model to learn multi-scale features without involving multiple networks (Shen et al., 2015) (Section 2.1.2).
3. We design a novel central pooling layer to retain much patch-center features rather than patch edge features. This strategy reserves much target-voxel-focused information and thereby achieved improved performance as opposed to uniformly distributed max pooling (Section 2.1.3).
4. During model training, we propose a sampling method to process imbalanced training labels and extract challenging patches to allow efficient model training. In this strategy, voxels are sampled where each voxel is assigned a weight score denoting its difficulty for segmentation (Section 2.3).

### 1.1. Related work

Approaches for lung nodule segmentation involved the detection of a Volume of Interest (VOI) covering the nodule area and segmentation inside this VOI. These methods can be generally classified into morphology methods (Diciotti et al., 2011; Messay et al., 2010), region growing methods, (Kubota et al., 2011; Song et al., 2016), energy optimization methods (Farag et al., 2013; Lassen et al., 2015), and machine-learning methods (Lu et al., 2013; Wu et al., 2010).

In morphology methods, morphological operations such as logic opening operation were applied for nodule-attached vessels removal (Kostis et al., 2003), then the connected component selection can separate lung nodules. However, the fixed-size morphological template is difficult to separate nodules that usually have wide contact surfaces with other anatomical objects. Consequently, more complex morphological operations that combine shape hypothesis were introduced. For instance, Kuhnigk et al. (2006) showed that the radius of vessels decreases while the vessels evolve along the periphery of the lungs. In addition, rolling ball filters (Messay et al., 2010) combined with rule-based analysis was also proposed for juxtapleural nodules. One notable difficulty for morphology methods is the morphological template size selection (Kubota et al., 2011), because it is difficult to find a suitable morphology template for various size of nodules. Non-solid nodules in particular are challenging for morphology operation (Diciotti et al., 2011).

In region growing methods, segmentation starts with a user-specified seed point, and voxels are included into nodule set iteratively until the pre-defined converge criterion is satisfied. These methods work well for isolated nodules. However, when analyzing juxtapleural nodules, region growing algorithm is known to be difficult to converge. Therefore, Dehmeshki et al. (2008) introduced a shape hypothesis and proposed sphericity contrast based region growing method to detach nodule from lung wall. Instead of using the current voxel intensity only, Kubota et al. (2011) constructed a probability map to denote the likelihood of each voxel belonging to nodule according to the local intensity value, then a region growing method was used to separate the nodule from background area. The common challenge for region growing methods is the converge criteria. Although shape constraint can be considered, irregular-shaped nodules remain difficult to process because the shape hypothesis can be violated.

In energy optimization methods, nodule segmentation is converted into an energy minimization task. The level-set-based methods, for example, use a level set function to describe the image, and the function is minimized when the segmented contour matches the nodule boundary (Chan and Vese, 2001). To detach nodules from lung wall, Farag et al. (2013) combined level set with shape prior hypothesis. In addition, graph cut algorithm (Boykov and Kolmogorov, 2004) was developed for lung nodule segmentation by framing the problem into a maximum flow optimization task. Ye et al. (2010) used a modification of the graph cut method. Such algorithm built an intensity and shape mode map through non-parametric mean shift clustering. Then, the graph cut algorithm was used for segmentation by using an energy formulation. However, similar to the region growing methods, the performance of these methods are typically adversely affected by juxtapleural nodules and low contrast nodules (e.g., GGO).

In machine-learning methods, researchers used classification models combined with high-level features for nodule segmentation (Lu et al., 2008; 2011). For instance, Wu et al. (2010) designed a set of texture and shape features to represent voxels. Afterwards, a conditional random field (CRF) model was trained for voxel classification. In addition, Lu et al. (2013) designed the spatial image features such that voxels of different nodule types were mapped into the same universal space. These high-level features were shown to be translation and rotational invariant.

As one of the data-driven methods, CNNs are conceptually similar to the previous machine-learning-based methods converting the segmentation task into voxel classification. A CNN model (Gao and Zhou, 2016; Shen et al., 2017) is a multi-layer neural network that learns hierarchical mappings between raw image data and labels. In medical image analysis, Ciresan et al. (2012) applied a CNN to neuronal membranes segmentation in electron microscopy images, where the segmentation task is converted into pixel classification. Also, Zhang et al. (2015) used a CNN model to segment brain matter in a voxel patch classification manner. In addition, CNN models using multi-view image patches (Prasoon et al., 2013) or multiple branches (Havaei et al., 2016) have been designed to extract features that are adaptive to different medical objects. On the other hand, fully convolutional neural networks (FCN) (Long et al., 2015) have been another trend for image segmentation. The FCN model involves up-sampling layers to make the output of CNN having the same size with the input image, and therefore requires only one forward propagation to segment the input image. For instance, Ronneberger et al. (2015) and Çiçek et al. (2016) proposed the U-Net model as a type of FCN approaches for biomedical image segmentation.

The major distinctions of the proposed CF-CNN model comparing to the previous approaches are three-fold: 1) we proposed a two-branch CNN architecture to learn both multi-view 3-D features and local texture features simultaneously; 2) we combined multiscale patches into a multi-channel patch that enables multi-scale feature extraction without involving multiple networks; 3) we proposed a novel central pooling layer to select features according to their spatial relevance to the target voxel (i.e., patch center voxel).

This paper is organized as follows. A detailed description of the proposed CF-CNN model is presented in Section 2. Experimental datasets and implementation details are introduced in Section 3. Section 4 provides the overall performance for the proposed method. Finally, Section 5 discusses the model design details and conclusion.

## 2. Methods

### 2.1. Model architecture

**Fig. 2:**
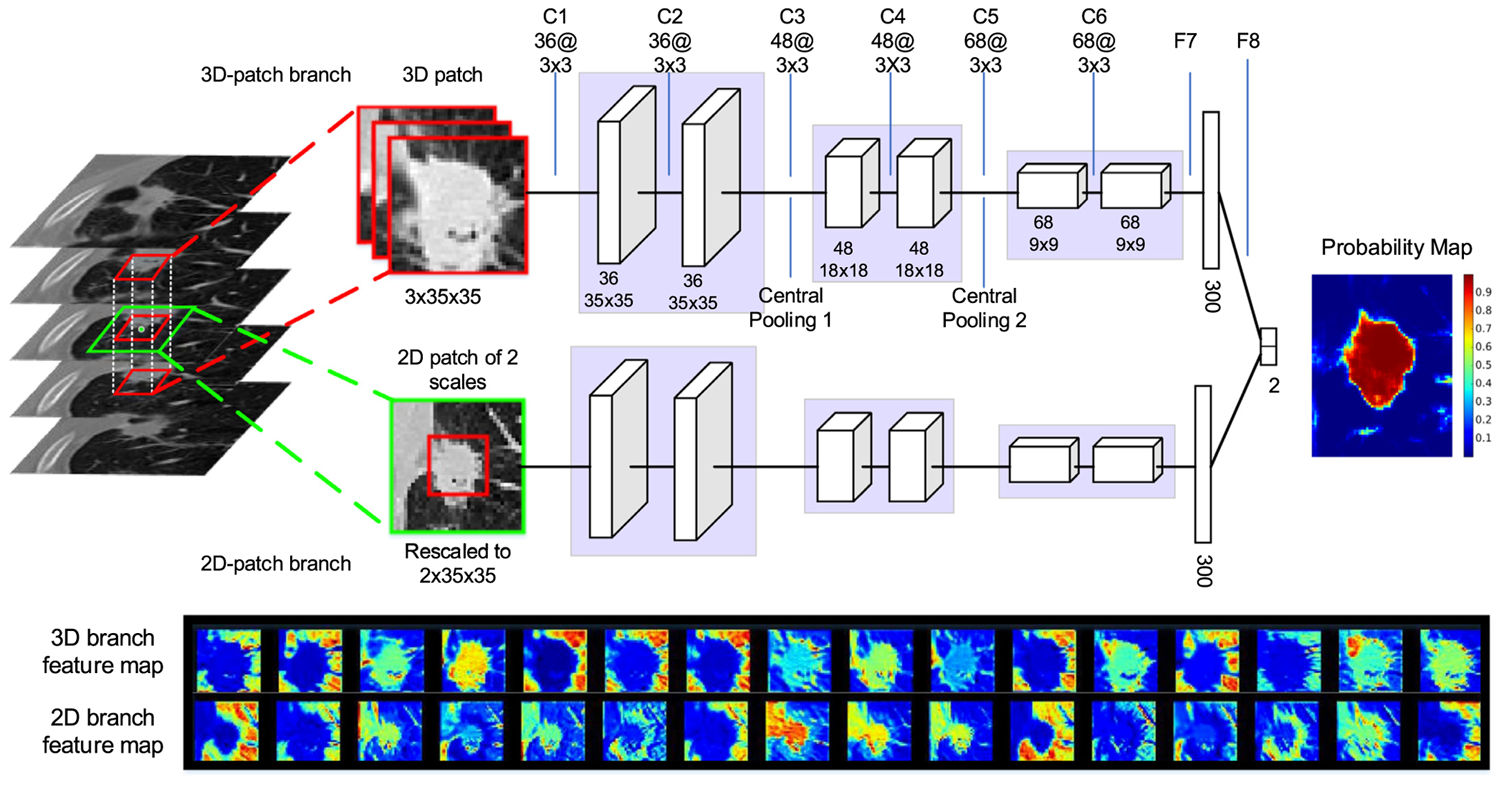
Illustration of the proposed CF-CNN architecture. The network contains six convolutional layers (C1–C6), two central pooling layers (central pooling 1 and central pooling 2), and two fully connected layers (F7, F8). The convolutional kernel size is denoted as *filter number* @ *filter width* × *filter height* (i.e., 36@3 × 3 represents 36 filters of kernel size 3 × 3). The number below each layer indicates the feature map size after convolution. After feeding all voxels into this CNN model, a probability map assigning each voxel the probability of it belonging to nodule is obtained. The bottom figure illustrates 16 randomly selected feature maps of the first convolutional layer (the first row is from the 3-D-patch CNN branch, the second row is from the 2-D-patch CNN branch). The feature maps indicate that the learned convolutional kernels can respond to various types of image characteristics such as edge or lung wall.

The proposed CF-CNN model utilizes 3-D and 2-D views of CT imaging for lung nodule segmentation (Fig. 2). Given one voxel in CT images, we extract a 3-D patch and a 2D multi-scale patch centered on this voxel as the input to the CNN model, and predict if this voxel belongs to the class of nodule or healthy tissues.

#### 2.1.1. CNN structure

The network includes two deep branches sharing the identical structure but are trained using different image patches. Each branch of the proposed CNN architecture consists of six convolutional layers, two central pooling layers (see detailed description in Section 2.1.3), and one fully connected layer. The six convolutional layers in this CNN are divided into three blocks, where each block shares the exact same structure including two convolutional layers of kernel size 3 × 3. These layers perform convolution operations on all input feature maps to obtain the output feature map defined by

(1) $$f^{j}=\mathrm{PReLU}\;\left(\sum_{i}c^{ij}*f^{i}+b^{j}\right)$$

where *f^i^* and *f^j^* are the *i*th input feature map and *j*th output feature map, respectively. We define *c^ij^* as the convolutional kernel between *f^i^* and *f^j^* (* denotes the 2-D convolution operation). *b^j^* is the bias of the *j*th output feature map. To accelerate training process, every convolutional layer is followed by batch normalization operation to normalize the corresponding output (Ioffe and Szegedy, 2015).

After each convolutional layer, a parametric rectified linear unit (PReLU) (He et al., 2015) is used as nonlinear activation function expressed as

(2) $$\mathrm{PReLU}\left(f^{j}\right)=\left\{ \begin{array}{ll} f^{j} & \text{if}\;f^{j}>0\\ a_{j}f^{j} & \text{if}\;f^{j}\leq 0 \end{array}\right.$$

In this equation, *a_j_* is a trainable parameter and *j* represents the *j*-th feature map in this convolutional layer. In our experiment, *a_j_* is initialized to be 0.25. The PReLU incorporates a non-zero slope controlled by the trainable parameter *a_j_* for negative inputs and has been proven to be more effective than the conventional ReLU (Krizhevsky et al., 2012) in ImageNet classification tasks. Between each block, we formulate a novel pooling method, termed central pooling, to select feature subsets from convolutional layers (more detailed description is provided in Section 2.1.3).

After the last convolutional layer (Fig. 2, C6), a fully connected layer is applied where each output unit connects to all inputs. This layer can capture correlations between different features produced by convolutional layer. For the purpose of achieving nonlinearity, PReLU is also used as an activation function after the fully connected layer. At the end of the model, the two CNN branches are combined by concatenating their fully connected layers (Fig. 2, F7). Finally, another fully connected layer (Fig. 2, F8) is applied to capture the correlations between the features from two CNN branches.

In the case of the output layer consisting of two units, the activation values are fed into a binary softmax function that are converted into probability distributions over the class labels. Namely, suppose that *o_k_* is the *k*th output of the network for a given input, the probability assigned to the *k*th class is the output of the softmax function:

(3) $$p_{k}=\exp(o_{k})/\sum_{h\subseteq \{0,1\}}\exp(o_{h})$$

where *k* = 0 and *k* = 1 represent non-nodule and nodule voxels respectively.

The goal of network training is to maximize the probability of the correct class. This is achieved by minimizing the cross-entropy loss for each training sample. Suppose that *y* is the true label for a given input patch that belongs to {0,1}, the loss function is defined as:

(4) $$L(W)=-\frac{1}{N}\sum_{n=1}^{N}\left[y_{n}\log\hat{y_{n}}+(1-y_{n})\log\;\left(1-\hat{y_{n}}\right)\right]+\lambda |W|$$

where $\hat{y_{n}}$ represents the predicted probability from CNN and *N* is the number of samples. To avoid over fitting, the 1 – *norm* regularization is used on the model weights *W. λ* controls the regularization strength, and is set to 5 × 10^−4^ in our model. The loss function is minimized during the model training process by computing the gradient of *L* over the network parameters *W.* During this process, the model weights *W* are initialized with the Xavier algorithm (Glorot and Bengio, 2010), and are updated using the stochastic gradient descent (SGD) algorithm (Havaei et al., 2016) as shown in Eq. (5).

(5) $$W_{t+1}=W_{t}+V_{t+1},\;V_{t+1}=\mu V_{t}-\alpha \;\nabla \;L(W_{t})$$

In this equation, *t* represents the training iteration number, and *V* is the update value initialized at zero. When calculating the gradient ∇*L*(*W*), only a batch of 128 samples are used, because it is difficult to store millions of training samples at memory one time (Krizhevsky et al., 2012). *μ* is the momentum that is set to 0.9 in our model. *α* is the learning rate which is updated using Eq. (6), and *α*_0_ is the base learning rate which is initialized to 6 × 10^−5^. *γ* and *p* are set to 0.0 0 01 and 0.75 respectively.

(6) $$\alpha_{t+1}=\alpha_{0}{\left(1+\gamma t\right)}^{-p}$$

#### 2.1.2. Two-branch architecture

The proposed two-branch network structure is designed to capture both 3-D and 2-D information simultaneously.

The 3-D-patch branch takes a 3-D volume of size 3 × 35 × 35 as input. Specifically, given one voxel, we extract a cuboid centered on this voxel that spreads the current, preceding and subsequent slices (see Fig. 2). This three-slice volume is treated as a three-channel image and is fed into the 3-D-patch CNN branch. Due to the large variance of CT image intensities, we normalize the three-channel patch using z-score that is defined as *f*(*x*) = (*x* – *x_mean_*)/*x_std_*. In this equation, *x_mean_* and *x_std_* represent the average and stand deviation of voxel intensities in the patch.

In parallel, we introduce a 2-D branch in attempt to focus on learning features from axial view images due to their high image resolution among all CT scans. We design the 2-D CNN branch to model the relationship between two scale patches jointly through convolutional layer. First, we extract two patches of size 65 × 65 and 35 × 35 on the target voxel. Then, we rescale them into the same size (35 × 35) using third-order spline interpolation to form a two-channel patch, and feed it into the 2-D CNN branch. The defined multi-scale patch strategy enables the model learning multiscale features within one network instead of training multiple separate networks.

#### 2.1.3. Central pooling

For a given image patch, it is intuitive that the voxels close to patch center are more relevant to the target voxel, whereas the patch edge voxels are less relevant. Therefore, we propose a central pooling operation to reserve many features around patch center as opposed to the traditional max pooling that has ignored the feature location information.

**Fig. 3:**
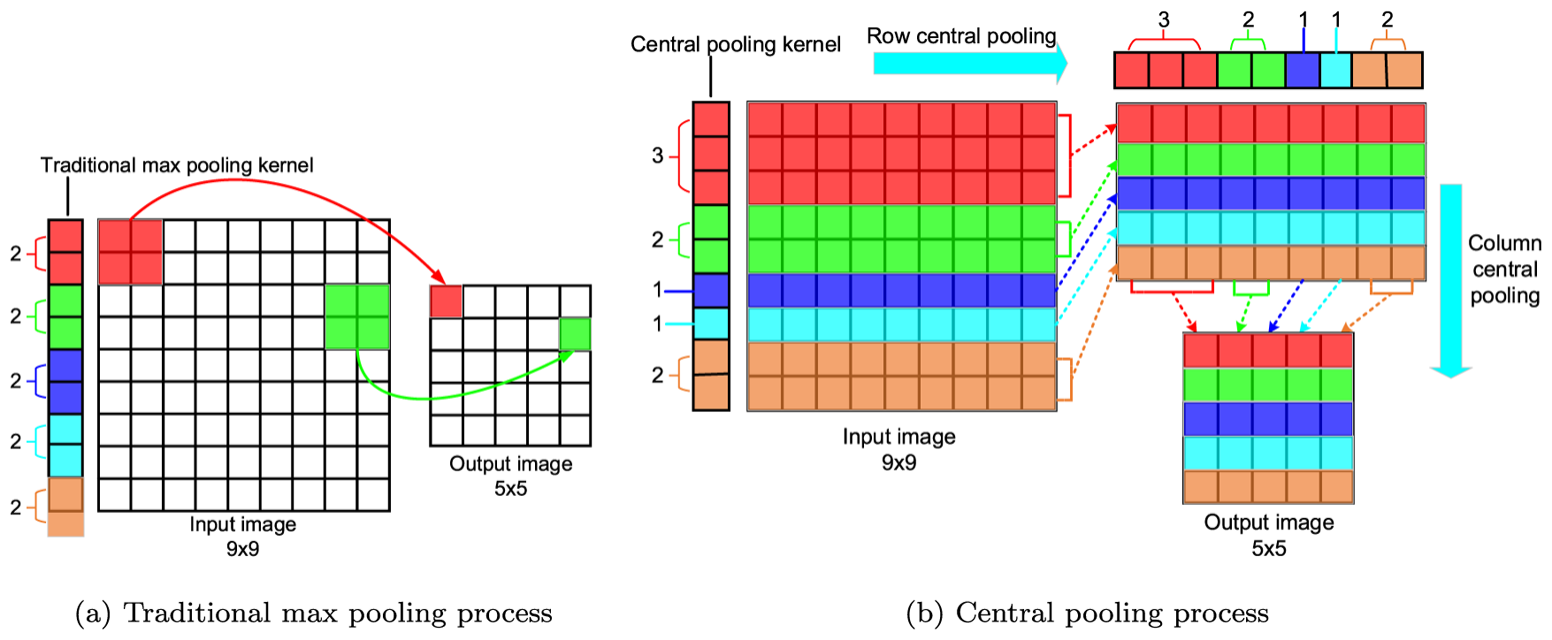
Central pooling process. (a) Traditional max pooling using 2 × 2 kernel and 2 voxel step. The pooling kernels are of the same size and are uniformly distributed. (b) Central pooling. The pooling kernel size varies according to the pooling position, and are non-uniformly distributed on input image. The pooling operation is firstly applied among rows, where we use small pooling kernels around image center, while large pooling kernels near image edge. Afterwards, we use the same pooling operation among columns. For the pooling kernel of size 2 and 3, the maximum value in a pooling window is selected as output.

Fig. 3(a) is the traditional max pooling operation where pooling kernels share the same size and are uniformly distributed on input image, while Fig. 3(b) illustrates the proposed central pooling process where the pooling kernel size varies according to the pooling position. In our design, we adopt small pooling kernels around image center and large pooling kernels around image edge. Since we intend to predict the label of the patch center voxel, the proposed central pooling is helpful to largely eliminate irrelevant patch edge features while retain patch center features at the same time.

This central pooling involves two parameters: 1) the size for different pooling kernel; 2) the number for each type of pooling kernel. In our work, we introduce three types of max pooling kernels (kernel size s = 1, 2, 3). The number for each type of kernels can be determined combining with the following rules: 1) we follow that the central pooling normally reduces the input image size by half on each axis as widely used in traditional max pooling (Havaei et al., 2016; Zhang et al., 2015); 2) to avoid large distortion caused by the non-uniformly distributed pooling kernel, we let half amount of all pooling kernels be 2 voxel size which is a common parameter used in traditional max pooling operation (Shen et al., 2017). After the number for three types of pooling kernels are determined, we symmetrically distribute all the kernels. For example, small pooling kernels (s = 1) are distributed around the image center, large kernels (s = 2, 3) are distributed close to the image edge symmetrically.

Given an input image of size *O* × *O,* the number *n*_1_, *n*_2_, and *n*_3_ for the three types of kernels can be determined using Eq. (7).

(7) $$\left\{ \begin{array}{l} n_{1}+2n_{2}+3n_{3}=O\\ n_{1}+n_{2}+n_{3}=O/2\\ n_{1}+n_{3}=n_{2} \end{array}\right.$$

The first equation ensures that the total length of all pooling kernels equals the input image size. The second equation denotes that after central pooling, the output image size is half of the input image size. The third equation ensures that half amount of pooling kernels are 2 voxel size. The unique solution of Eq. (7) is that *n*_1_ = *O*/8, *n*_2_ = *O*/4, and *n*_3_ = *O*/8. However, *O* may not be divisible by 8 or 4. We solve this problem in two steps: 1) first, we let *n*_1_ = ⌊*O*/8⌋, *n*_2_ = ⌊*O*/4⌋, and *n*_3_ = ⌊*O*/8⌋, where ⌊ · ⌋ denotes the rounding down operation. After this operation, there is a residual *r* ∈ [0, 7] left (produced by *O*/8 – ⌊*O*/8⌋); 2) then, we build a look-up table *L* (Table 1) to assign the number for the three types of kernels to cover the *r* voxels. Finally, *n*_1_, *n*_2_, and *n*_3_ are determined using Eq. (8)

(8) $$\left\{ \begin{array}{l} n_{1}=\left\lfloor O/8\right\rfloor +L[1,r]\\ n_{2}=\left\lfloor O/4\right\rfloor +L[2,r]\\ n_{3}=\left\lfloor O/8\right\rfloor +L[3,r] \end{array}\right.$$

In Eq. (8), *L*[*i, r*] represents the value at the *i*th row and the *r*th column of the look-up table. For instance, when the input image is 9 × 9, then *n*_1_ = 1 + 1 = 2, *n*_2_ = 2 + 0 = 2, *n*_3_ = 1 + 0 = 1. Afterwards, these kernels are symmetrically distributed on the input image, which is in the kernel size order of {3, 2, 1, 1, 2} for this case. Since 2-D pooling kernels of different size cannot be distributed continuously on the input image, we use 1-D pooling kernel to do row pooling first and column pooling afterwards. This central pooling process is illustrated in Fig. 3(b).

**Fig. 4:**
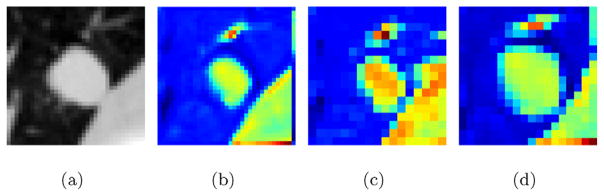
Traditional max pooling and central pooling comparison. (a) Data patch. (b) A feature map from the first convolutional layer in CF-CNN model (Fig 2). (c) Traditional max pooling of the feature map. (d) Central pooling of the feature map. The color in this figure represents the voxel values in the feature map. Red denotes big value and blue represents small value. (For interpretation of the references to color in this figure legend, the reader is referred to the web version of this article.)

For a better understanding of the difference between central pooling and traditional max pooling, Fig. 4 shows the pooling results for one convolutional feature map using these two pooling methods. The central pooling reserves more information around the feature map center compared with traditional max pooling.

### 2.2. 3-D processing

**Fig. 5:**
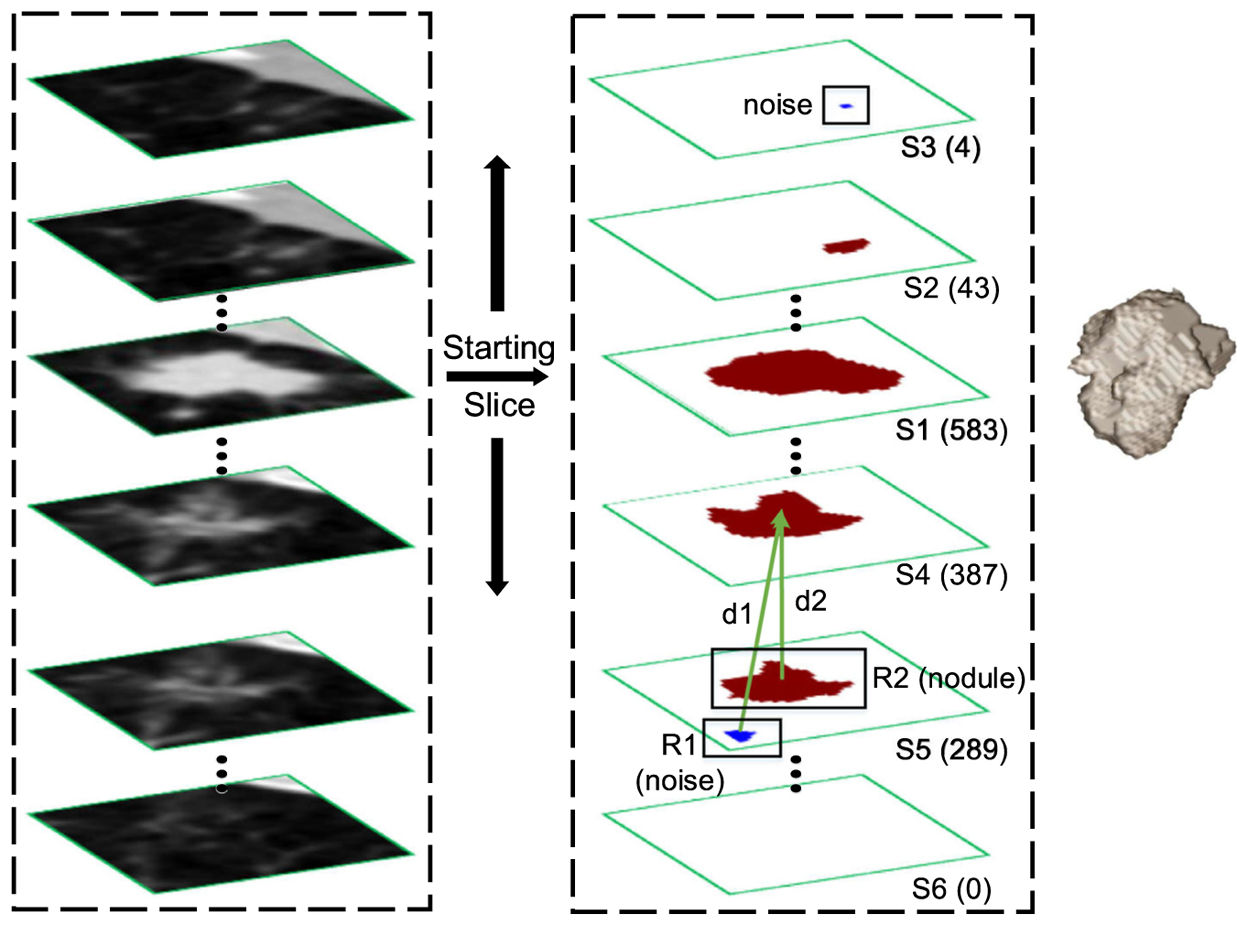
Three-dimensional segmentation procedure. The bounding box is only specified on one slice designated the starting slice, and then applied to the preceding and following slices iteratively. The number on the right side of each slice is the area of the nodule counted in voxels. The column on the left displays the original CT slices and the middle column shows the outcome of the CF-CNN model, where the red and blue regions represent nodules and false positive noises, respectively (the latter one is earmarked for removal). The image on the right is the 3-D visualization of the ultimate segmentation outcome. (For interpretation of the references to color in this figure legend, the reader is referred to the web version of this article.)

To initialize the proposed CF-CNN model, a bounding cuboid for the nodule is specified to enable voxel classification within such cuboid. Because a nodule is typically spread over multiple slices, the process of manually specifying bounding cuboid is tedious. We overcome this problem by only specifying a bounding box on one slice which is called the starting slice (S1 in Fig. 5).

The same bounding box is then applied to the preceding and subsequent slice repeatedly until at least one of the following two experimental conditions are satisfied: 1) no segmented nodule voxel exists in this slice (Fig. 5, slice S6) or 2) the nodule area in this slice is less than 30% of the nodule area in the preceding slice. For instance, slice S3 in Fig. 5 is eliminated because the segmented nodule only contains four voxels, which is only 10% of the size of the preceding slice (slice S2). To remove noisy voxels such as isolated tiny regions during 3-D process (blue R1 in slice S5), we made a simple connected component selection as following: 1) when the noise arises in the starting slice, we select the isolated region that is closest to the bounding box center. 2) when the noise arises in other slices, we select the isolated region whose massive center is closest to the massive center in the nodule of the preceding slice. For instance, two segmented candidate regions R1 and R2 are generated by the CF-CNN in slice S5. The distances between the massive center in these two regions and that of the preceding nodule (slice S4) are denoted as d1 and d2. Since d2 *<* d1, region R2 is reserved and R1(noise) is discarded.

### 2.3. Training sample selection

**Fig. 6:**
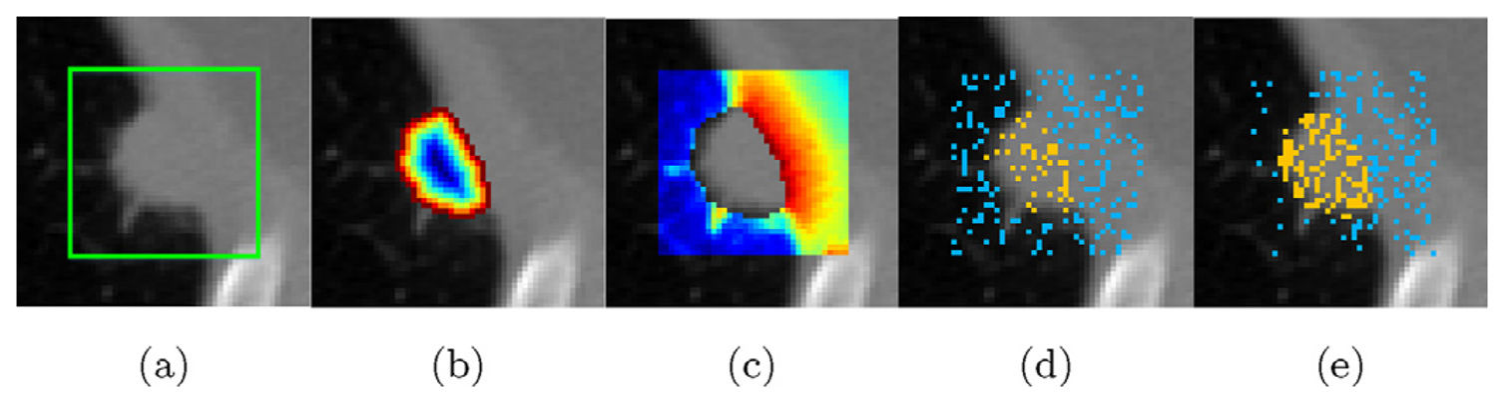
Illustration of weighted sampling process. (a) A CT image in training set. The green box is acquired by expanding eight voxels on each axis of the nodule bounding box. (b) nodule voxel weight distribution. (c) non-nodule voxel weight distribution. (d) random sampling result. (e) weighted sampling result. Yellow and blue crosses denote sampled nodule and non-nodule voxels, respectively. (For interpretation of the references to color in this figure legend, the reader is referred to the web version of this article.)

Since our method focuses on learning CNN-based features from images automatically, large amount of voxel patches (as training samples) are greatly needed to facilitate the model training. However, lung nodule segmentation is a highly data imbalanced problem where nodule voxels usually counted less than 5% of the total voxels in one CT slice. Selecting training voxel patches randomly would easily cause model to be overwhelmed by non-nodule features. Therefore, we propose a weighted sampling strategy to select only part of the whole voxel patches according to their degree of segmentation difficulty. First, we identify the nodule bounding box for each CT slice in training set, and expand eight voxels on each axis to get an expanded box (the green box in Fig. 6(a)). Then, for each voxel inside this expanded box, we assign them a weight score indicating their segmentation difficulty. Finally, 40% nodule voxel patches and the same amount of non-nodule voxel patches are sampled according to their corresponding weight score. In this process, we sample nodule and non-nodule voxel patches separately to balance the training labels.

When choosing nodule patches, we intend to sample more nodule edge patches rather than nodule center patches, because nodule edges typically contain more texture information for segmentation. Consequently, our goal of finding challenging nodule voxels is converted into finding nodule edge voxels. This process can be formulated by assigning each nodule voxel *i* a weight *PW_i_* using the distance function defined in Eq. (9).

(9) $$PW_{i}=\exp\;\left(-\min_{j\subseteq N}d(i,j)\right)/Z$$

In this equation, *N* is the non-nodule voxel set, and *d* (*i, j*) is the euclidean distance between nodule voxel *i* and non-nodule voxel *j. Z* is a normalization factor to make the weight of all voxels accumulated to 1. *PW_i_* is a number between [0, 1] which demonstrates the weight of the current nodule voxel being sampled. Fig. 6(b) visualizes the nodule voxel weight distribution of one nodule slice (Fig 6(a)) using this method.

When choosing non-nodule patches, the challenge voxels are from nodule-attached lung wall and vessels. We identify these voxels from two considerable aspects: 1) we utilize a distance function to assign each voxel a weight which decreases as the voxel apart from nodule area. 2) we intend to eliminate the dark lung field area, since they usually have very low intensity and can be easily distinguished from nodule voxels. This process is formulated by assigning each non-nodule voxel *i* a weight *NW_i_* using Eq. (10),

(10) $$NW_{i}=I_{i}\exp\;\left(-\min_{j\subseteq P}d(i,j)\right)/Z$$

where *P* is the nodule voxel set, and *d* (*i, j*) is the euclidean distance between non-nodule voxel *i* and nodule voxel *j.* Since the dark lung field area can also get a high response similar to lung wall, we multiply the normalized CT image intensity *I_i_* to the *exp* () function to eliminate the dark lung field area. Fig. 6(c) illustrates the weight distribution for non-nodule voxels.

Finally, 40% nodule voxels were sampled according to *PW_i_,* and the same amount of non-nodule voxels were sampled according *NW_i_*. Compared with random sampling (Fig. 6(d)), our strategy (Fig. 6(e)) selected more nodule edge and nodule-attached lung wall voxels which were particularly useful for training CNN models.

## 3. Data and experiment

### 3.1. Data

We used two datasets in our experimental evaluation. The first one is a publicly available dataset from the Lung Image Database Consortium and Image Database Resource Initiative (LIDC). The second dataset is independently collected from Guangdong General Hospital (GDGH).

#### LIDC dataset

The dataset contains CT images of 2610 lung nodules from seven academic centers and eight medical imaging companies around the world (Armato et al., 2011; Setio et al., 2016). Nodule diameters in this dataset range from 2.03 mm to 38.12 mm, and the slice interval ranges from 0.45 mm to 5.0 mm. The axial plan resolution varies from 0.46 mm × 0.46 mm to 0.98 mm × 0.98 mm. All the nodules are annotated by up to four board-certified radiologists. In this work, we studied nodule samples that are annotated with available four radiologists (a total of 893 nodules). Because of the inter-variability among four different radiologists, a 50% consensus criterion (Kubota et al., 2011) is adopted to generate a ground-truth boundary. For all the 893 selected nodules, each expert has been asked to independently assess multiple subjective clinical characteristics including sphericity, spiculation, and the likelihood of malignancy (Armato et al., 2011).

We randomly partitioned the 893 nodules into three subsets including training, validation and testing sets that are comprised of 350, 50 and 493 nodules respectively. As seen in Table 2, the three subsets share similar statistical distributions of clinical characteristics. We train the CF-CNN model only on the training set, and the validation set is used for determining the CNN training epoch number. Finally, the testing set is used for performance evaluation.

#### GDGH dataset

The second dataset from Guangdong General Hospital consists of 74 patients with single nodules. Nodule diameters range from 1.64 mm to 58.94 mm with the average diameters (mean ± standard deviation) of 25.79 ± 12.47 mm. The CT slice interval varies from 1.25 mm to 2.5 mm with the axial plan resolution ranging from 0.61 mm × 0.61 mm to 0.88 mm × 0.88 mm. All nodules were annotated by an experienced radiologist and verified by another radiologist (10+ years experience) in thoracic imaging of lung lesions. To further validate the segmentation performance, after obtaining the trained CF-CNN model on LIDC dataset, we directly evaluate the segmentation results by testing on this independent nodule set.

### 3.2. Evaluation criteria

Given the ground truth segmentation *Gt* and automated segmentation result *Auto,* the dice similarity coefficient (DSC) and symmetric average surface distance (ASD) are used as the primary evaluation criteria for assessing the automatic segmentation accuracy. DSC is a widely used metric for measuring the overlap between two segmentation results (Havaei et al., 2016; Valverde et al., 2017), and ASD measures the average boundary distance between surfaces of two segmentation results (Gao et al., 2016). In addition, we also use the sensitivity (SEN) and positive predictive value (PPV) to demonstrate the voxel classification accuracy (Gao et al., 2016). Full definitions are listed as in Eq. (11) to Eq. (13) :

(11) $$\mathrm{DSC}=\frac{2\cdot V(Gt\cap \mathrm{Auto})}{V(Gt)+V(\mathrm{Auto})}$$

(12) $$\mathrm{ASD}=\frac{1}{2}\left({\text{mean}}_{i\in Gt}\min_{j\in \mathrm{Auto}}d(i,j)+{\text{mean}}_{i\in \mathrm{Auto}}\min_{j\in Gt}d(i,j)\right)$$

(13) $$\mathrm{SEN}=\frac{V(Gt\cap \mathrm{Auto})}{V(Gt)},\mathrm{PPV}=\frac{V(Gt\cap \mathrm{Auto})}{V(\mathrm{Auto})}$$

where *V* is the volume size counted in voxels and *d*(*i,j*) denotes the Euclidean distance between voxel *i* and voxel *j* measured in millimeters.

### 3.3. Implementation details

In our experiment, we generated 0.41 million voxel patches on the LIDC training set using the weighted sampling method (Section 2.3). When each training epoch was completed, the CF-CNN model was tested on the validation set and evaluated by the DSC value. After 21 epochs of training, the DSC on validation set became stable, therefore, we decided to train the CF-CNN model for 21 epochs. Finally, the model performance was evaluated on the independent testing set. Our method was implemented in Python 2.7 and all experiments were performed on a machine with an Intel Core i7-4790K CPU and 8GB memory. The CNN was implemented using *CAFFE* Toolkit (Jia et al., 2014) and was accelerated on an NVIDIA GTX-980Ti GPU (6GB on-board memory). The CF-CNN model converged after 9 h of training on 0.41 million voxel patches.

Two widely used methods including level set with active contours (Chan and Vese, 2001) and graph cut (Boykov and Kolmogorov, 2004) were used as comparison with the proposed CF-CNN. The parameters in level set and graph cut were all optimized by a parameter grid searching using the *Fiji* software (Schindelin et al., 2012). In the level set method, fast marching was firstly used to generate the initial nodule contour. Then, active contours model was used for further contour refining. The parameters were set as: gray value threshold = 50, distance threshold = 0.1 for fast marching; and advection = 2.20, curvature = 1.00, gray scale tolerance = 30.00, convergence = 0.005 for active contours model. In the graph cut method, there were two parameters involved in *Fiji* software. These parameters were set as: data prior (foreground bias) = 0.86 and edge weights (smoothness) = 0.56. Both the level set and graph cut methods were applied on 2-D image slices. Finally, the same slices were selected for measuring performance across all compared methods.

Furthermore, to compare the proposed CF-CNN with other deep-learning methods, we implemented a state-of-the-art FCN model, termed U-Net (Ronneberger et al., 2015). The network architecture we used was the same with the published paper. To make a fair comparison, all the training images were from the nodule ROI image instead of the whole CT scan. Since the input image size of U-Net was 572 × 572, we padded the nodule ROI image with “reflect padding” to fit the 572 × 572 size, meaning that the padded voxels were acquired by mirroring the existing image. In the testing phase, we used the ground-truth bounding box for initialization of all the compared methods (e.g., level set, graph cut and U-Net) to ensure a fair experimental comparison, and they were all post-processed using the same way.

## 4. Results

### 4.1. Overall performance

**Fig. 7:**
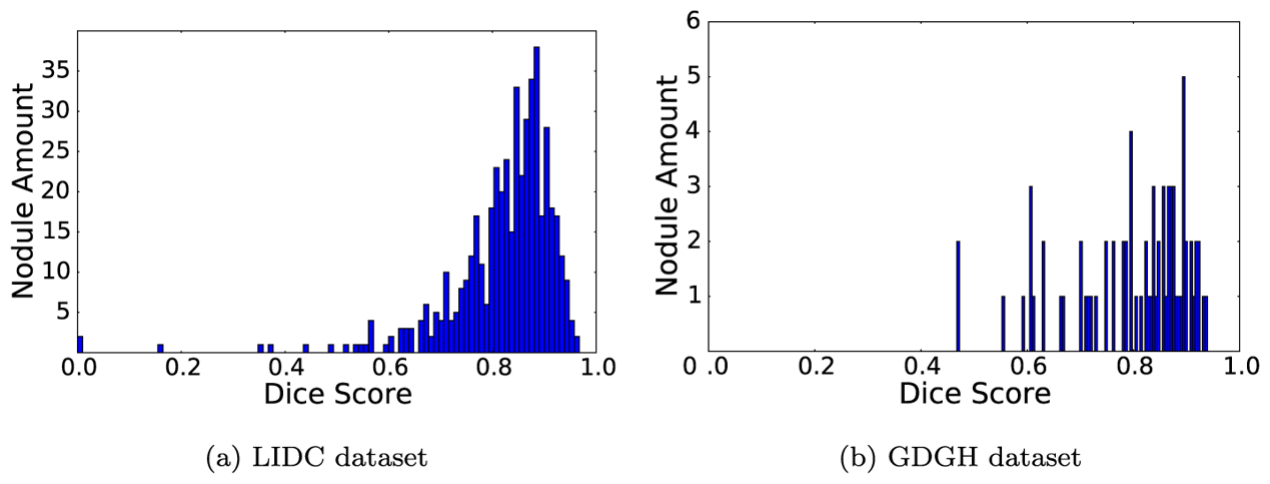
DSC distributions of the LIDC testing set and GDGH dataset.

From Table 3, we observed that the proposed CF-CNN model outperformed graph cut and level set on LIDC dataset. In addition, when testing on the independent GDGH dataset, the strong results of CF-CNN reaffirmed the competitive outcomes of segmenting different types of lung nodules. In particular, we demonstrated the advantage of the proposed central pooling layer by additionally comparing CF-CNN with CF-CNN-MP, where CF-CNN-MP represents the CF-CNN model with traditional max pooling. Table 3 illustrated that the proposed central pooling layer improved the average DSC value by around 2% on both datasets. In addition, the combination of 3-D-patch and 2-D-patch branches also improved the model performance. CF-CNN outperformed single 3-D-patch branch or single 2-D-patch branch as indicated in Table 3. To enable a full observation of all testing nodules, as seen in Fig. 7, we showed the overall distributions of the obtained DSC scores from the two datasets.

In Table 3, the ground truth was combined with the annotations by four radiologists using a 50% consistency criterion. To have an intuition of the consistency between different individual human experts, we performed a pairwise DSC comparison between the CF-CNN and the four radiologists as demonstrated in Table 4. Our results showed that the DSC between CF-CNN and each radiologist is 81.66% on average, which compared favorably with the average inter-radiologist variability of 83.64%. Moreover, the CF-CNN also showed stability when compared with four different radiologists, since the average DSC between CF-CNN and each radiologist is stable at 81.57%–81.72%.

As mentioned in Section 1.1, Kubota et al. (2011), Messay et al. (2010) and other researchers also evaluated their methods on the LIDC dataset. Consequently, in Table 5, we listed the methods which required only a user initialization (e.g., a VOI or seed point) procedure without further user interaction. Since these methods used overlap *O* = *V* (*Gt* ∩ *Auto*)/*V* (*Gt* ∪ *Auto*) to measure the model performance, we additionally reported our results using the same measurement in this table. Of note, among all the listed methods, our method is evaluated on a larger amount of testing set including different types of lung nodules as presented in Fig. 1.

### 4.2. Robustness of segmentation

We showed that CF-CNN can process various types of nodules with similar performance indicating potential segmentation robustness. The LIDC testing set includes various nodules reflecting different levels of segmentation difficulties. All the nodules in this dataset were given nine characteristics to represent their sphericity, the likelihood of malignancy, and other properties. We chose four representative characteristics and divided the testing set into different groups according to their characteristic scores. The average DSCs on different groups are shown in Table 6. All groups in this table have similar DSC values which highlighted that our approach was able to capture nodule shapes with regards to different clinical characteristics.

In Table 7, we further summarized results of challenging attached nodules (juxtapleural and juxtavascular nodules). The proposed CF-CNN presented appealing performance on these nodules. The outcomes indicated a potential robustness of CF-CNN segmentation that was irrespective of attached conditions of nodules.

### 4.3. Visualization

**Fig. 8:**
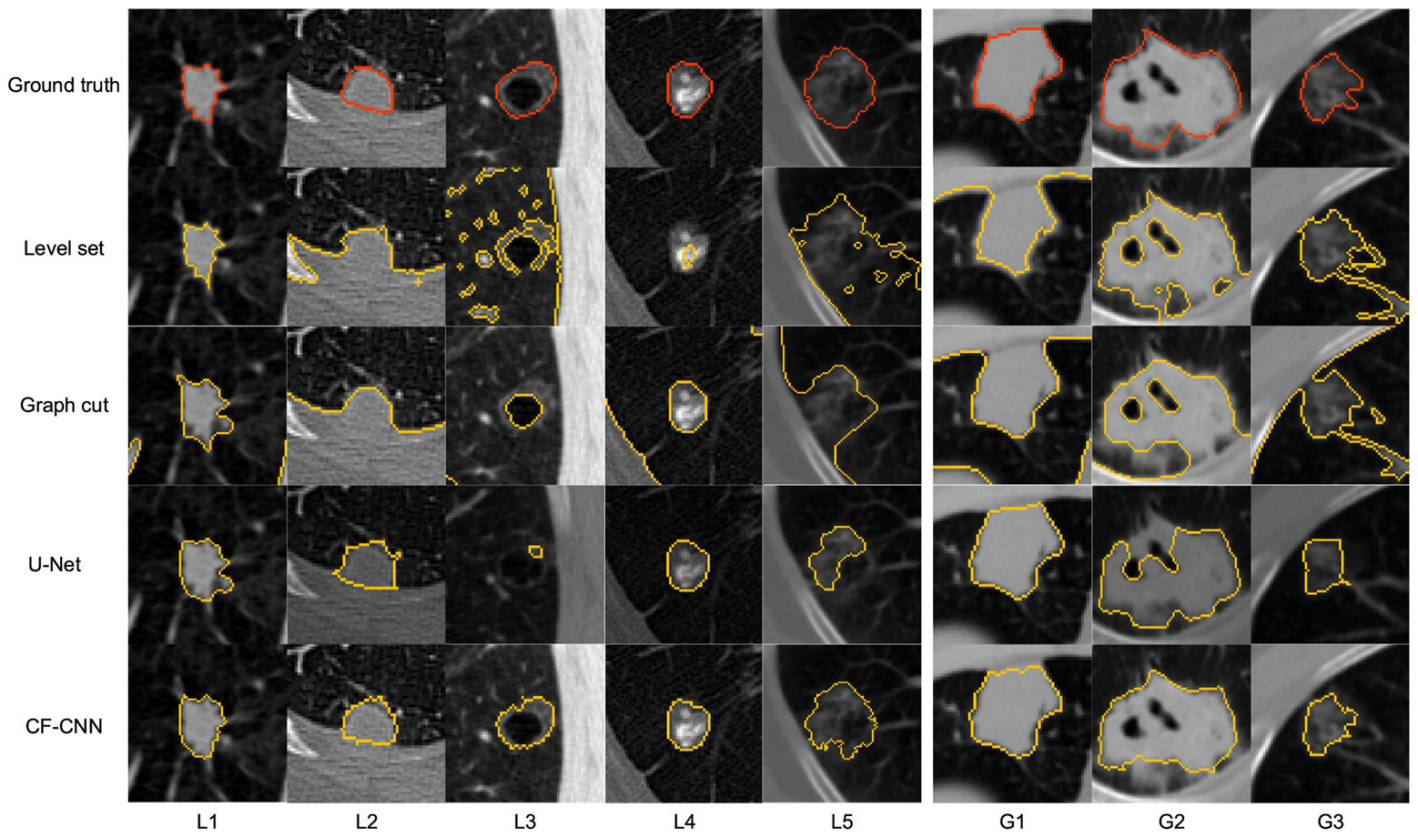
Segmentation results visualization. From top to bottom: nodule with ground truth, level set segmentation, graph cut segmentation, U-Net segmentation, and CF-CNN segmentation. L1-L5 are nodules of different types from the LIDC testing set. G1-G3 are nodules from the GDGH dataset.

The segmentation results were visualized to allow comparison of different approaches. We demonstrated five representative nodules from the LIDC testing set (Fig. 8, L1-L5) and three challenging nodules from the GDGH dataset (Fig. 8, G1-G3).

For isolated solid nodules (L1), both our method and the state-of-the-art methods performed well. However, when examining nodules attached to surrounding tissues (L2), the level set and graph cut methods reduced performance because they were struggling to differentiate nodules from pleura. In contrast, the proposed CF-CNN remained robust when segmenting such nodules, showing the good feature learning ability of the CF-CNN model. For cavitary nodules (L3), the level set, graph cut, and U-Net methods falsely considered the cavity region as background; however, CF-CNN was able to reserve it correctly. Because of the heterogeneous intensity contrast between calcific and non-calcific tissues, the level set method identified only the calcific region whereas ignored the non-calcific part. In contrast, our CF-CNN was able to detect both parts and reserve the complete nodule (L4). When examining GGO nodules containing cavity structures inside (L5), level set and graph cut methods tended to show under segmentation, since they cannot distinguish nodule voxels from background because of the low intensity contrast. Affected by the cavity structure, outputs from U-Net identified only part of the nodule (L5).

When testing on the GDGH dataset, challenging nodules were mostly found to be juxtapleural and multi-cavitary. Fig. 8(G1-G3) showed the segmentation results of different methods on these challenging cases in the GDGH dataset. G1, G2, and G3 indicated juxtapleural, multi-cavitary, and GGO nodule attached to vessel respectively. When attachment happened between nodule and lung wall (G1) or vessel (G3), the level set and graph cut methods intended to show under segmentation, because they were unable to identify attached tissues from nodule. Similar to the L3 nodule in LIDC testing set, the cavity structures also affected the U-Net performance (G2) in the GDGH dataset. Whereas CF-CNN performed reasonably well in capturing the complete nodule shape.

**Fig. 9:**
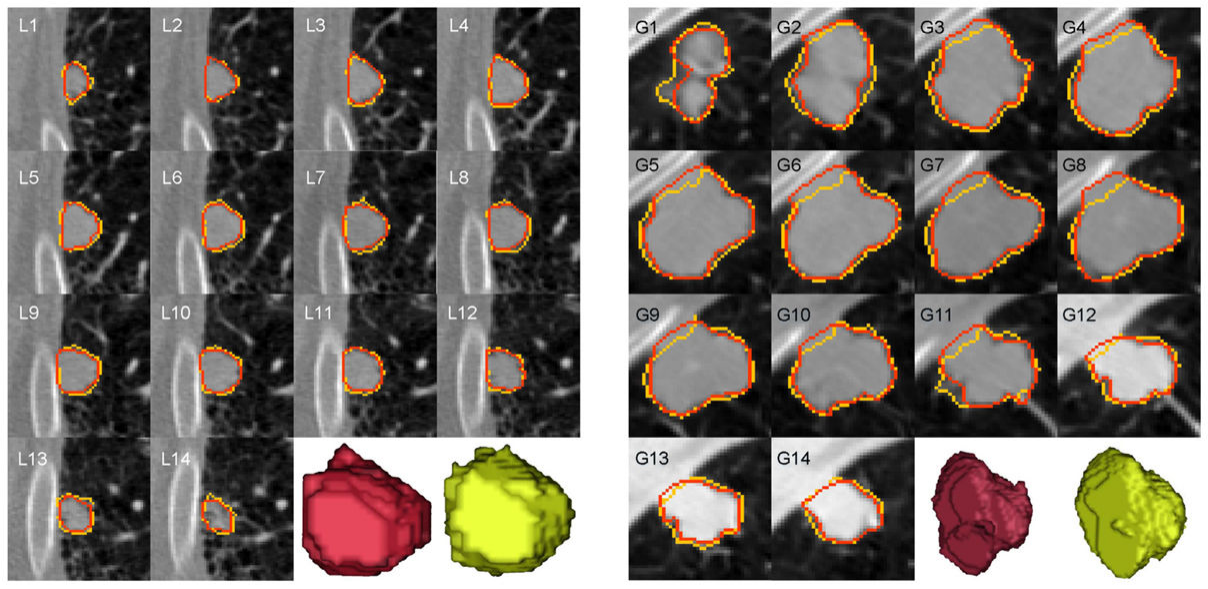
Segmentation results of CF-CNN on two juxtapleural nodules from the LIDC testing set (L1-L14) and GDGH dataset (G1-G14). The red and yellow contours denote the ground truth and the segmentation results of the CF-CNN method, respectively. The numbers in the upper left corner of each image represent the CT slice number of this nodule. The red and yellow 3-D renderings are from the ground truth and the CF-CNN results. (For interpretation of the references to color in this figure legend, the reader is referred to the web version of this article.)

Fig. 9 further showed multiple segmented slices of two juxtapleural cases from the LIDC testing set and GDGH dataset when applying CF-CNN. This figure indicated that the segmentation results of CF-CNN showed much overlap with the ground truth contours.

## 5. Discussion and conclusion

In this study, we proposed a CF-CNN model for lung nodule segmentation that leveraged both 2-D and 3-D volumetric CT images. The approach demonstrated a strong ability to learn nodule-sensitive features automatically from large amounts of CT image patches (0.41 million voxel patches). Our CF-CNN achieved encouraging segmentation accuracy on nodules with various clinical characteristics (Table 6). By comparing with several widely used lung nodule segmentation methods, our method showed superior performance in segmentation accuracy (DSC = 82.15% for LIDC and DSC = 80.02% for GDGH, Table 3). Especially, the CF-CNN model can successfully segment challenging cases where nodules were attached to pleura (Table 7). Moreover, we compared our results with the inter-radiologists consistency on LIDC dataset, showing a difference in average dice score of only 1.98% (Table 4).

The CF-CNN model specifically incorporated a two-branch structure for extracting both 3-D and 2-D features simultaneously. The 3-D-patch branch can learn features from different image views, while the 2-D branch extracted multi-scale features through a multi-scale patch strategy. For a given voxel patch, the patch center textures were more relevant to the target voxel. Consequently, we proposed a novel central pooling layer to reserve much patch center features while eliminate redundant patch edge features. This operation applied non-uniformly distributed pooling kernels on the input image. Specifically, small pooling kernels (size s = 1) are distributed around patch center while large pooling kernels (s = 2, 3) are located around input patch edge.

In the design of convolutional architecture, we adopted deep stacked structure with small convolutional kernels (size 3 × 3) instead of a shallow structure with big convolutional kernels. In fact, a stack of two 3 × 3 convolutional kernels have an effective respective field of 5 × 5 (Simonyan and Zisserman, 2014). However, the stacked two-layer structure allows the extraction of stronger nonlinear deep features benefiting from the design of PReLU activation function after each layer (Lin et al., 2013). In addition, small convolutional kernels are known to be able to reduce the parameter numbers in the network, thereby making it less prone to over fitting (Simonyan and Zisserman, 2014).

During model training, the training voxel patch selection is difficult because of the imbalanced labels. Specifically, only less than 5% voxels belong to nodule while more than 95% voxels are non-nodule tissues in training CT images. We solved this problem by proposing a weighted sampling method to select only the challenging voxels. In practice, nodule edge voxels and nodule-attached voxels are found to be challenging for various segmentation methods. Consequently, in the training set, we defined two distance functions to find these voxels, and assigned them higher weight score than other voxels. Finally, 40% of all voxels were sampled according to their corresponding weight scores.

Since the proposed CF-CNN is particularly dealing with volumetric CT imaging data at scale, we incorporated a GPU accelerator to gain computational efficiency. When segmenting a nodule in a 50 × 50 image, the CF-CNN only required 6.92 seconds after GPU acceleration. Furthermore, the use of specified bounding box largely reduced the computational burden in searching the nodule space in CT. It is the only pre-procedure that allowed our CF-CNN to initialize the computation. Alternatively, dedicated lung nodule detection algorithms providing an estimated cuboid of nodule can be directly integrated into our method to construct a fully automatic system.

In conclusion, the proposed CF-CNN model highlighted the power of image-based deep learning architecture in finding discriminative features for lung nodule segmentation. Our approach presented a unique advantage in capturing nodule-sensitive information from CT imaging data. In the future work, we plan to integrate our proposed central pooling layer and two-branch architecture into the FCN network to seek potential improvement and reduce computational burdens.

## Figures and Tables

**Table 1:** Look-up table *L*.

Kernel size	Residual *r*							

	0	1	2	3	4	5	6	7
s= 1	0	1	0	1	0	0	0	0
s= 2	0	0	1	1	2	1	3	2
s= 3	0	0	0	0	0	1	0	1

*Note:* Given a residual *r* ∈ [0, 7], this table assigns the number for the three types of kernels (s = 1, 2, 3) such that they can cover the *r* voxels.

**Table 2:** Characteristic distributions of the LIDC training, validation and testing sets. Values are shown in mean ± standard deviation.

Characteristics	Training set (n = 350)	Validation set (n = 50)	Testing set (n = 493)
Diameter (mm)	9.48± 4.89	9.21± 5.03	9.35± 4.88
Spiculation	1.72± 0.86	1.65± 0.74	1.71± 0.87
Lobulation	1.87± 0.81	1.81± 0.69	1.82± 0.81
Sphericity	3.84± 0.62	3.79± 0.63	3.86± 0.59
Calcification	5.67± 0.78	5.57± 0.90	5.63± 0.87
Malignancy	3.05± 0.91	3.01± 1.04	4.15± 0.97

*Note:* All characteristic values except diameter and calcification are on ordinal scale of 1–5, while calcification value ranges from 2 to 6. Spiculation and lobulation represent the amount of these shapes that present in one nodule. Sphericity, calcification, and malignancy represent the likelihood of these characteristics in one nodule. The characteristics on three sets are without significant statistical difference.

**Table 3:** Mean ± standard deviation of quantitative results for various segmentation methods. The best performance is indicated in bold font.

LIDC Set	DSC (%)	ASD (mm)	SEN (%)	PPV (%)
Level Set	60.63± 17.39	0.48± 0.25	64.38± 22.75	71.03± 24.35
Graph Cut	68.90± 16.03	0.48± 0.30	80.81± 15.25	65.09± 22.42
U-Net	79.50± 13.95	0.24± 0.23	86.81± 18.43	78.18± 16.13
3-D-Patch Branch	79.20± 11.88	0.21± 0.17	90.93± 14.72	72.91± 13.73
2-D-Patch Branch	80.47± 11.23	0.18± 0.15	91.36± 14.40	74.64± 13.16
CF-CNN-MP	80.39± 11.90	0.18± 0.15	91.33± 14.88	74.52± 13.54
CF-CNN	**82.15**± **10.76**	**0.17**± **0.23**	**92.75**± **12.83**	**75.84**± **13.14**

GDGH Set	DSC (%)	ASD (mm)	SEN (%)	PPV (%)

Level Set	66.02± 17.21	0.78± 0.65	60.83± 17.98	79.24± 21.38
Graph Cut	74.13± 13.32	0.83± 0.56	82.94± 13.66	69.24± 16.60
U-Net	75.26± 11.82	0.49 ± 0.48	76.65± 16.42	77.21± 11.57
3-D-Patch Branch	77.89± 10.64	0.40± 0.31	81.29± 15.60	76.95± 11.62
2-D-Patch Branch	78.98± 11.96	0.38± 0.39	81.42± 16.90	**79.65**± **12.20**
CF-CNN-MP	78.61± 12.18	0.39± 0.38	80.93± 17.07	79.38± 12.03
CF-CNN	**80.02**± **11.09**	**0.35**± **0.34**	**83.19**± **15.22**	79.30± 12.09

*Note:* 3-D-Patch Branch and 2-D-Patch Branch represent the 3-D and 2-D branches in CF-CNN model. CF-CNN-MP represents the CF-CNN model using traditional max pooling instead of central pooling.

**Table 4:** Mean DSCs (%) of pair-wise comparison between each radiologist and CF-CNN. R1 to R4 represent the four radiologists.

	R1	R2	R3	R4	Average
R2	83.45	–	83.76	83.98	
R3	83.32	83.76	–	83.61	**83.64** ± **0.25**
R4	83.25	83.98	83.61	–	
CF-CNN	81.72	81.66	81.57	81.67	**81.66** ± **0.05**

**Table 5:** Performance of various lung nodule segmentation methods on the LIDC-IDRI dataset.

Methods	Year	Nodule amount		Overlap

		Training	Testing	
Tachibana and Kido (2006)	2006	–	23	50.7 ± 21.9%
Wang et al. (2009)	2009	23	64	58%
Messay et al. (2010)	2010	–	68	63 ± 16%
Kubota et al. (2011)	2011	–	23	69 ± 18%
			82	59 ± 19%
Tan et al. (2013)	2013	–	23	65%
Lassen et al. (2015)	2015	–	19	52 ± 7%
			40	50 ± 14%
Messay et al. (2015)	2015	300	66	71.70 ± 19.89%
			77	69.23 ± 13.82%
**Proposed CF-CNN**	**2017**	**350**	**493**	**71.16**± **12.22**%

**Table 6:** DSCs on different nodule groups of the LIDC testing set.

Characteristics	Characteristic scores					

	1	2	3	4	5	6
Spiculation	81.49	82.43	82.03	84.57	85.95	–
	[239]	[185]	[27]	[37]	[5]	–
Malignancy	82.40	78.30	81.36	84.73	87.81	–
	[46]	[95]	[183]	[149]	[20]	–
Calcification	–	88.64	83.28	83.05	86.15	81.77
	–	[2]	[26]	[36]	[20]	[409]
Sphericity	–	78.12	79.92	82.66	83.47	–
	–	[10]	[90]	[344]	[49]	–

*Note:* The nodules are grouped based on their clinical characteristic scores. The numbers in square brackets represent the number of nodules in this group. The characteristic scores given by four radiologists are averaged.

**Table 7:** DSCs and ASDs for attached and non-attached nodules on the two testing sets.

	LIDC testing set		GDGH dataset	
	
	Attached (n = 113)	Non-attached (n = 380)	Attached (n = 18)	Non-attached (n = 56)
DSC (%)	81.65	82.30	80.55	79.85
ASD (mm)	0.21	0.16	0.37	0.35
